# A malignant choroid plexus tumour with prevailing immature blastematous elements

**DOI:** 10.1111/nan.12764

**Published:** 2021-09-15

**Authors:** Arnault Tauziède‐Espariat, Mélanie Pagès, Julien Masliah‐Planchon, Franck Bourdeaut, François Doz, Kévin Beccaria, Nathalie Boddaert, Lauren Hasty, Emmanuèle Lechapt, Christian Thomas, Werner Paulus, Pascale Varlet, Martin Hasselblatt

**Affiliations:** ^1^ Department of Neuropathology GHU Paris‐Neurosciences, Sainte‐Anne Hospital Paris France; ^2^ Institut Curie Paris Sciences Lettres University, SIREDO, INSERM U830, Laboratory of Translational Research in Paediatric Oncology Paris France; ^3^ Department of Genetic Curie Institute Paris France; ^4^ Institut Curie SIREDO (Care, Innovation, Research in Pediatric, Adolescent and Young Adults Oncology) Paris France; ^5^ Université de Paris Paris France; ^6^ Department of Paediatric Neurosurgery Necker Hospital, APHP, Université Paris Descartes Paris France; ^7^ Department of Radiology Necker Hospital, APHP, Université Paris Descartes Paris France; ^8^ Institute of Neuropathology University Hospital Münster Münster Germany

**Keywords:** blastematous, choroid plexus tumour, DNA methylation profiling, mesenchymal

## INTRODUCTION

Choroid plexus tumours are intraventricular neoplasms derived from the choroid plexus epithelium. In contrast to choroid plexus papilloma, choroid plexus carcinoma shows frank signs of malignancy and is associated with infancy, *TP53* alterations and an aggressive clinical course.^1^ DNA methylation profiling segregates choroid plexus tumours into three distinct epigenetic subgroups: supratentorial paediatric low‐risk choroid plexus tumours, infratentorial adult low‐risk choroid plexus tumours and supratentorial paediatric high‐risk choroid plexus tumours (subclass paediatric B).^2^, ^3^, ^4^ Choroid plexus tumours may acquire unusual histological features (e.g. oncocytic change, mucinous degeneration, melanisation, tubular/glandular architecture, neuropil‐like islands, bone, cartilage or adipose tissue formation) and may also show unstructured solid growth.^5^ A malignant choroid plexus tumour with prevailing blastematous features, however, has not yet to our knowledge been reported.

**FIGURE 1:**
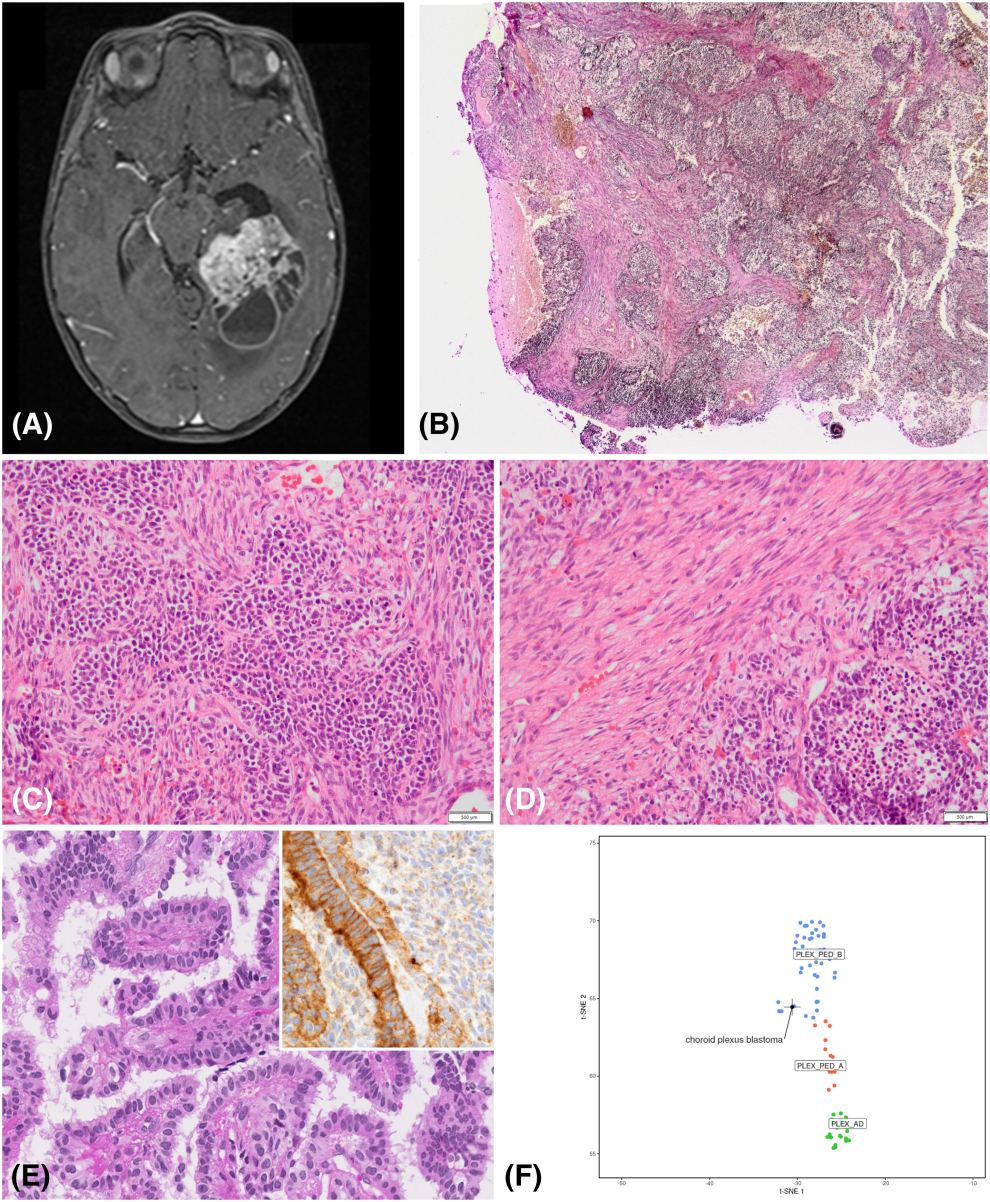
Radiological, histopathological and molecular findings. Magnetic resonance imaging shows a large intraventricular tumour with heterogeneous contrast enhancement (A). Histopathology of the highly cellular tumour showing a pleomorphic mesenchymal component rich in connective tissue and elastic material with transitions to immature blastematous cells arranged in nests (B, haematoxylin, phloxin and saffron staining, magnification ×60; C and D, haematoxylin, phloxin and saffron staining, magnification ×400). Only focally, a papillary growth pattern reminiscent of the non‐neoplastic choroid plexus (Figure 1E) and expression of choroid plexus marker Kir7.1 (inset) is encountered. TSNE analysis confirms similarity with the molecular subgroup ‘choroid plexus tumour, subclass paediatric B’. (F) Black scale bars represent 500 μm for panel (B) and 50 μm for panels (C)–(E)

A 3‐year‐old girl, with a family history of neuroblastoma (first cousin), suffered from intracranial hypertension due to a large tumour located within the left lateral ventricle showing heterogeneous contrast enhancement (Figure 1A). Gross total resection was achieved. Upon histopathological examination, the highly cellular tumour displayed a pleomorphic spindle cell mesenchymal component rich in connective tissue and elastic material with transitions to immature poorly differentiated blastematous cells arranged in nests (Figure 1B–D). Focally, there was a papillary growth pattern reminiscent of the non‐neoplastic choroid plexus (Figure 1E). Mitotic activity was elevated, and microvascular proliferation and tumour necrosis were present. Using immunohistochemical staining, the tumour cells displayed polyphenotypic differentiation with positivity for cytokeratins (MNF116) and vimentin, but also focal staining for GFAP, synaptophysin, MITF, HMB45, smooth muscle actin and desmin (Figure S1). Expressions of CK18 and membranous staining of the choroid plexus tumour marker Kir7.1 were encountered in areas of focal papillary growth but gradually lost in tumour cells showing a less differentiated phenotype (inset Figure 1E). Staining for SALL4, EMA, myogenin, Olig2, p53, Lin28A, NUT, BCOR and germinal markers (OCT3/4, PLAP, beta‐HCG and alpha‐fetoprotein) were negative. Silver impregnation stains showed the reticulin network in the tumour was abundant. Nuclear expression of SMARCB1/INI1 and SMARCA4/Brg1 was retained (normal). The Ki67/MIB‐labelling index was 70% in the poorly differentiated component. Custom panel sequencing (including *TP53*, *DICER1*, *DROSHA* and *DGCR8* genes) performed on germline and tumour DNA did not reveal any pathogenic alterations. Using RNAseq (TruSeq RNA Library Prep; Illumina) and a set of fusion detection tools (FusionMAP, FusionCatcher, STAR‐fusion and Defuse), no fusion transcripts could be identified. Copy number profiling showed gains of chromosome 2, 7, 8,12, 19 and 20 and a more focal amplification of 300 Kb on chromosome 20q11.2 containing the genes *XKR7*, *CCM2L*, *HCK*, *TM9SF4* and *PLAGL2* (Figure S2).

DNA‐methylation profiling (Illumina Methylation EPIC BeadChip) using the Heidelberg central nervous system (CNS) tumour classifier^3^ showed the case to be a ‘choroid plexus tumour, subclass paediatric B’ (calibrated score 0.99), and the similarity with this molecular subgroup was further confirmed by t‐SNE analysis (Figure 1F). Based on the histopathological features of this malignant choroid plexus tumour, a diagnosis of choroid plexus tumour with immature blastematous elements was established. The patient received conventional and high‐dose chemotherapy followed by proton therapy. Seventeen months after the initial resection, a distant recurrence affecting the right parietal lobe was detected on follow‐up imaging and subsequently confirmed by biopsy.

Blastomas are malignant childhood neoplasms composed of immature elements, which are thought to recapitulate early stages of organ development. A solid growth pattern with mixed layers showing mesenchymal and blastematous differentiation is the hallmark feature of pleuropulmonary blastomas but has also been described in blastomas found in other locations. The histopathological similarity of the present case showing immature blastematous and mesenchymal elements could justify a diagnosis of choroid plexus blastoma.

As in the majority of choroid plexus tumours, genetic events that drive the development of choroid plexus tumour with immature blastematous elements remain to be determined.^6^ The absence of somatic and germline *DICER1* mutations in the present case argues against a link with *DICER1*‐associated entities such as pituitary blastoma, pineoblastoma, *DICER1*‐associated CNS sarcoma and embryonal tumour with multilayered rosettes.^7^, ^8^

DNA methylation profiles represent a combination of both somatically acquired DNA methylation changes and a signature reflecting the cell of origin. The high similarity of the DNA methylation profile with choroid plexus tumours of the molecular subclass paediatric B is characteristic for malignant choroid plexus tumours and may suggest that the tumour secondarily acquired blastematous features. Among choroid plexus tumours from our archives (including 102 cases of the molecular subclass paediatric B), no further cases with immature blastematous elements were encountered, suggesting that immature blastematous elements are not a characteristic feature of molecular subclass paediatric B tumours. Clinical history, immunohistochemical staining profile and molecular findings argue not only against the possibility of other CNS tumour entities but also against metastatic disease, which is frequent in pleuropulmonary blastoma patients.^9^ We had initially also considered the possibility of teratoma, but the histopathological features would be unusual, and the DNA methylation profiling results make this possibility unlikely.

To conclude, malignant choroid tumours may show prevailing immature blastematous elements, that show similarities with blastomas found in other locations. This case also highlights the value of DNA‐methylation profiling in the diagnosis of choroid plexus tumours with unusual histopathological features.

## ETHICS STATEMENT

This study was approved by the GHU Paris Psychiatry Neurosciences, Sainte‐Anne Hospital's local ethics committee. The patient signed informed consent forms.

## CONFLICT OF INTERESTS

The authors declare that they have no conflict of interest directly related to the topic of this article.

## AUTHOR CONTRIBUTIONS

A.T.‐E., M.P., J M‐P., F.B., F.D., K.B., N.B., L.H., E.L., C.T., W.P., P.V. and M.H. were involved in the acquisition, analysis or interpretation of data. A.T.‐E. and M.H. drafted the manuscript, and all co‐authors revised it critically for intellectual content.

### PEER REVIEW

The peer review history for this article is available at https://publons.com/publon/10.1111/nan.12764.

### DATA AVAILABILITY STATEMENT

The data that support the findings of this study are available from the corresponding authors upon reasonable request.

## Supporting information


**Figure S1.** **Immunohistochemistry.** Tumour cells show polyphenotypic differentiation with positivity for cytokeratins [MNF116, and CK18 (the latter predominantly in areas reminiscent of the non‐neoplastic choroid plexus)], but also focal staining for synaptophysin, GFAP, actin, desmin, HMB45, MITF and diffuse staining for vimentin. Presence of reticulin fibres using silver impregnation. The Ki67/MIB‐labelling index accounts for 70% in the poorly differentiated component.

**Figure S2.** **Copy number variation profile.** Copy number variation analysis showed several aneuploidies without any amplification.
