# Supplementary figures and images for: A malignant choroid plexus tumour with prevailing immature blastematous elements
Source: Neuropathol Appl Neurobiol. 2021 Sep 15;48(2):e12764. doi: 10.1111/nan.12764 (PMC9292497; doi:10.1111/nan.12764)

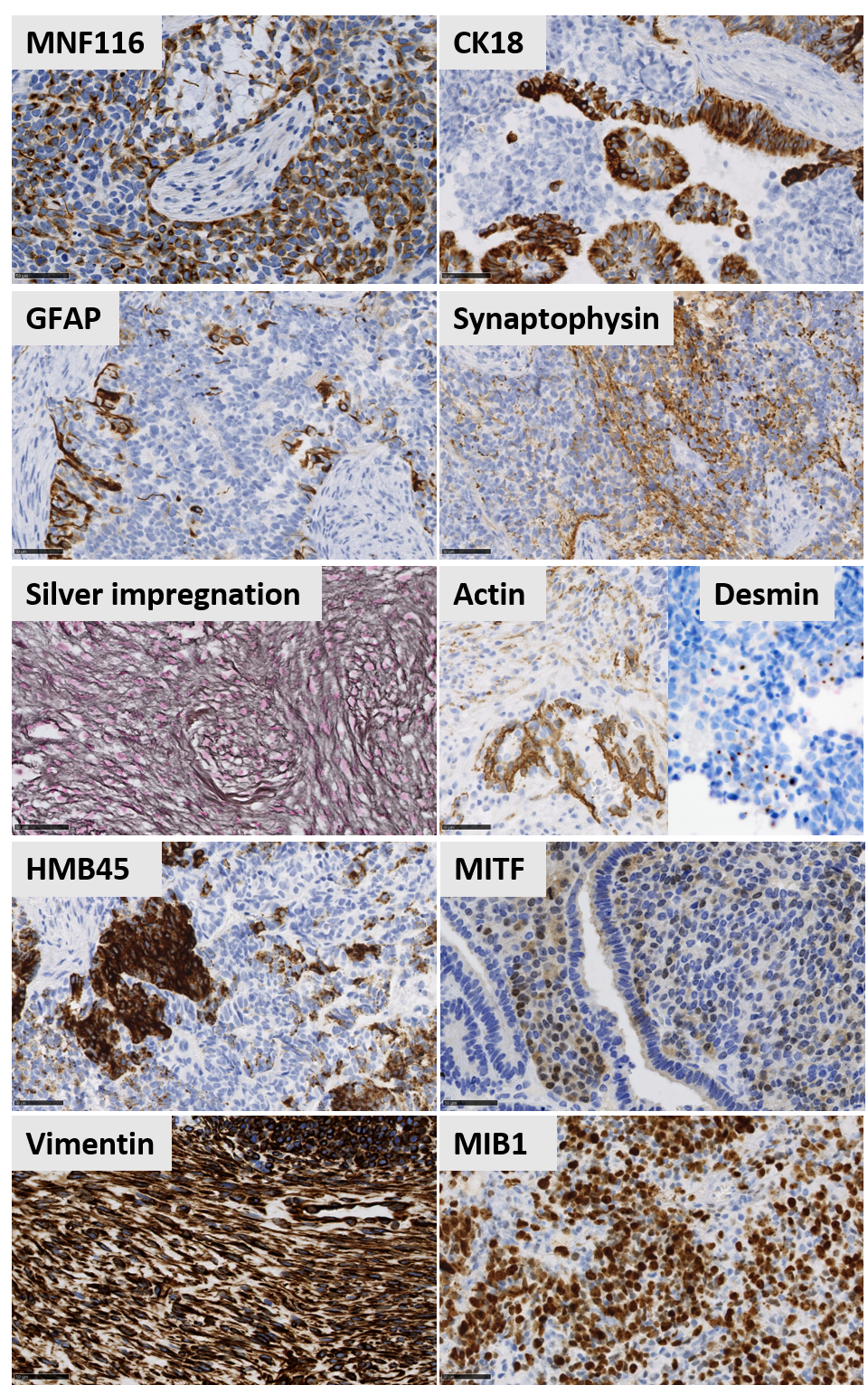

Supplement: Supplementary file 1 — Figure S1. Immunohistochemistry. Tumour cells show polyphenotypic differentiation with positivity for cytokeratins [MNF116, and CK18 (the latter predominantly in areas reminiscent of the non‐neoplastic choroid plexus)], but also focal staining for synaptophysin, GFAP, actin, desmin, HMB45, MITF and diffuse staining for vimentin. Presence of reticulin fibres using silver impregnation. The Ki67/MIB‐labelling index accounts for 70% in the poorly differentiated component. [file NAN-48-0-s002.tif]

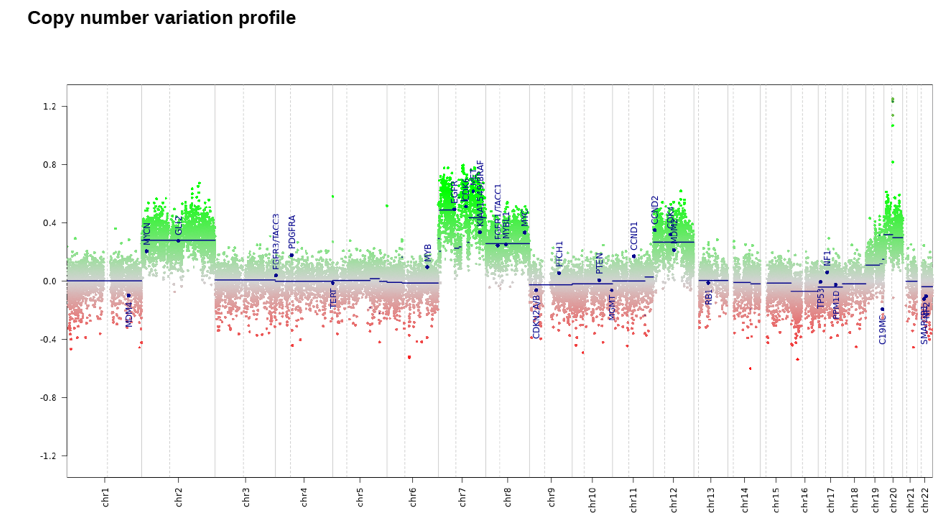

Supplement: Supplementary file 2 — Figure S2. Copy number variation profile. Copy number variation analysis showed several aneuploidies without any amplification. [file NAN-48-0-s001.tif]
